# Impact of broiler breeder growth trajectory on plasma corticosterone concentration: a comparison of analytical methods

**DOI:** 10.1016/j.psj.2022.101792

**Published:** 2022-02-23

**Authors:** Mohammad Afrouziyeh, Martin J. Zuidhof

**Affiliations:** Department of Agricultural, Food and Nutritional Science, University of Alberta, Edmonton, AB, Canada T6G 2P5

**Keywords:** broiler breeder, corticosterone, ELISA, metabolomics, feed restriction

## Abstract

Blood concentration of corticosterone (**CORT**) is a measure of welfare in feed restricted broiler breeders. The RIA and ELISA have been routinely used for measuring CORT in blood, excreta, and feather. Due to the presence of some confounding factors in the aforementioned colorimetric enzyme reaction methods, some methodological difficulties have been attributed to those assays. The correlation between broiler breeder plasma CORT concentrations, measured using ELISA and a novel liquid chromatography-tandem mass spectrometry (**LC-MS/MS**) method, was the focus of the current study. A total of 36 broiler breeder pullets were used, of which 30 were randomly assigned to one of 10 unique growth trajectories, and 6 were assigned to an unrestricted group. We designed the growth trajectories using a 3-phase Gompertz growth model with 10 levels of BW gain in the prepubertal and pubertal growth phases, ranging from the breeder-recommended target BW (**CON**) to 22.5% higher (CON+22.5%), in 2.5% increments. The BW trajectories were applied to each individual bird using a precision feeding (**PF**) system, which collected BW and feed intake data. The birds were classified based on age at first egg (**AFE**), and 12 birds each having the highest and lowest AFE was selected for the CORT study. Then median photostimulation BW of the candidate birds was used to define the upper (heavy BW) and lower (standard BW) extremes, and plasma CORT levels were evaluated by ELISA and LC-MS/MS methods from their blood collected at 18, 20, 22, 24, and 26 wk of age. Concentrations of plasma CORT measured using ELISA method were highly correlated (r = 0.95; *P* < 0.001) with values measured using LC-MS/MS method, validating interchangeably usage of both methods to measure plasma CORT in broiler breeders. Plasma CORT levels were not affected by photostimulation BW or breeders' age, indicating same welfare status between the precision fed high and low BW groups.

## Introduction

Numerous studies have shown that high levels of corticosterone (**CORT**) are associated with greater stress level in severely feed restricted broiler breeders. Stressors activate the hypothalamus-pituitary-adrenocortical cascade, resulting in the release of CORT (Blas, 2015). Much effort through experimental studies has been devoted to quantifying and characterizing this association and the underlying mechanisms. In most studies RIA and ELISA methods have been used for measuring CORT in the blood and feather (Carbajal et al., 2014; Cognuck et al., 2020; Gonzales et al., 2003; Häffelin et al., 2020; Leishman et al., 2020; Weimer et al., 2020).

ELISA assay employs a colorimetric enzyme reaction which can be confounded by many factors. The method requires the avoidance of contact with light and metal, precise control of the amount of acid added in sample analysis, and a long detection time, leading to a lack of proper validation testing (Little et al., 2008; Shu et al., 2003; Sink et al., 2008; Zhong and Suo, 1998; Zhou et al., 2011). Stanczyk et al. (2007) described that due to lack of standardization across steroid hormone assays (either RIA or ELISA), it is difficult to compare results across studies that use different assay platforms. Mouse plasma CORT level was measured using an ultra-fast liquid chromatography-tandem mass spectrometry (**LC-MS/MS**) method (Huan et al., 2014), but the correlation of the measures with ELISA method has remained to be elucidated. Thus, it is prudent to determine the degree in which CORT concentration measured using the classic ELISA is correlated with LC-MS/MS measure.

Broiler breeders are commonly reared by applying a substantial reduction in feed intake that controls excessive growth and maximizes reproductive fitness, production persistency, and longevity (Renema and Robinson, 2004). Feed restriction programs can be categorized into quantitative (e.g., limited everyday feed restriction and skip-a-day feeding programs) and qualitative (e.g., feeding diluted diets) feed restriction programs (Carneiro et al., 2019; Zuidhof et al., 1995). Selection for increased growth rate in broilers has led to an increase in adult BW for their parent stocks (Zuidhof et al., 2014). However, broiler breeder BW targets have changed very little over the past decades (Renema et al., 2007), creating a considerable gap between growth potential of broilers and broiler breeder target BW. Over decades, reducing feed consumption to control breeder BW has increased the intensity of feed restriction, causing welfare concerns in underfed birds (van Krimpen and de Jong, 2014). The welfare aspect of different feeding programs has been assessed through physiological indices of stress such as elevated blood heterophil:lymphocyte ratio, plasma content CORT, cecal content CORT, colon content CORT, feather content CORT (de Jong et al., 2002, 2005; Hocking et al., 2001; Mormėde et al., 2007; van Krimpen and de Jong, 2014; Weimer et al., 2018) and behavioral changes such as stereotypic object pecking, over-drinking, and hyperactivity (de Jong and Jones, 2006). Weimer et al. (2018) compared multiple abovementioned stress indicators to elucidate the correlation between the measures in broiler chickens. The authors concluded that although it is prudent to measure multiple physiological indices of stress to assess the animal welfare aspect of feeding programs, blood CORT was found to be the most reliable measure of stress, showing less variation than other measures.

To reduce the intensity of feed restriction in broiler breeders, we investigated the effects of incremental increases in target BW gain, including a nonrestricted group, during prepubertal and pubertal growth phases on feeding motivation and reproductive performance (Zukiwsky et al., 2021). In the current study, we have evaluated the effect of the high and low photostimulation BW on plasma CORT level as an index of welfare. The objectives of this study were to 1) determine correlation between plasma concentrations of CORT measured by ELISA and LC-MS/MS methods and 2) investigate the effects of the high and low photostimulation BW and breeder age on plasma CORT levels.

## Materials and Methods

The animal protocol for the study was approved by the University of Alberta Animal Care and Use Committee for Livestock and followed the Canadian Council on Animal Care guidelines and policies (CCAC, 2009).

### Animals and Management

The detailed experimental protocol was published previously (Zukiwsky et al., 2021). Briefly, Ross 708 pullets (n = 36) were placed in a single pen containing 2 precision feeding (**PF**) stations, from hatch to 43 wk of age at a stocking density of 3.0 birds per m^2^. The birds were fed commercial broiler breeder diets: starter (crumble; ME 2,726 kcal/kg, 21% CP, 1.00% Ca, and 0.45% available P) from hatch to d 34; grower (mash; ME 2,799 kcal/kg, 15% CP, 0.79% Ca, and 0.44% available P) from d 35 to d 179; and laying diet (crumble; ME 2,798 kcal/kg, 15.3% CP, 3.30% Ca, and 0.38% available P) from d 180 onward. Feed was provided through the PF system, which identified individual birds using a wing band containing a radio frequency identification (**RFID**) transponder and fed them according to how their real-time BW compared to the preprogrammed target BW (Zuidhof et al., 2019). All birds had access to the PF stations 24 h per day throughout the experiment. The PF system provided several access to a small meal for a 60 s time period if the individual bird real-time BW was equal or less than its preprogrammed target BW; otherwise, the system gently ejected the birds from the PF station. All birds had ad libitum access to water throughout the experiment.

Pullets were exposed to 8L:16D (15 lx) photoschedule during the rearing phase and were photostimulated at wk 22 by increasing the photoperiod to 11L:13D (20 lx); to 12L:12D (25 lx) on wk 23, then at wk 24 to 13L:11D (50 lx) for the remainder of the experiment. A trap nest with 8 nesting sites and a nest box with 8 nesting sites equipped with RFID readers, which identified and weighed eggs of individual hens, were installed in the room at 14 wk of age, which allowed pullets to adapt to the nesting system prior to the onset of lay.

### Experimental Design, Photostimulation BW, and Age at First Egg

**Figure 1:**
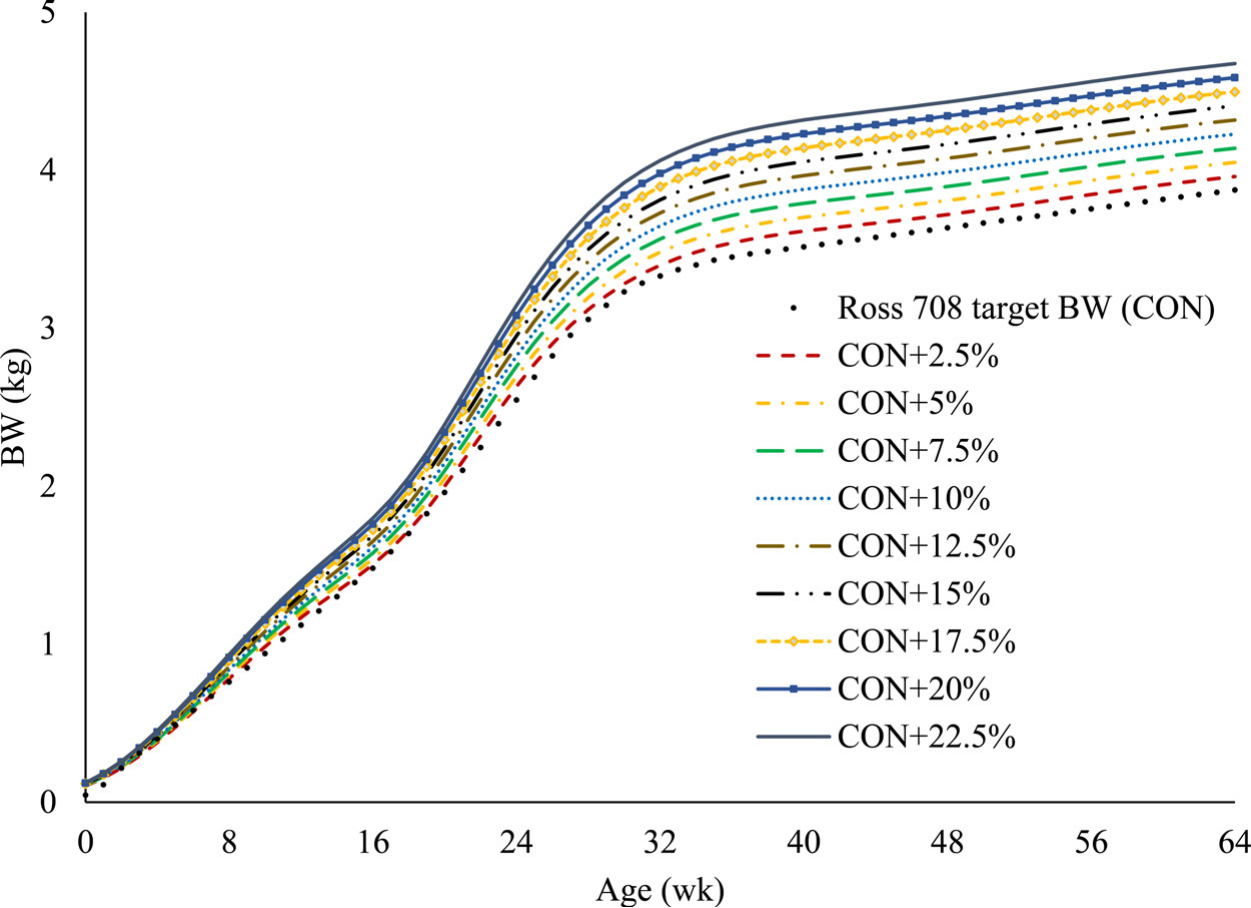
Growth trajectories designed using estimated coefficients of a 3 phase Gompertz model. General model form was BW_t_ = $\sum_{i=1}^{i=3}g_{i}exp^{-exp^{-b_{i}\left(t-I_{i}\right)}}$.where BW_t_ was BW (kg) at time t (wk); i was thgrowth phase (i = 1 to 3) where phase 1, 2, and 3 corresponded roughly to prepubertal, pubertal, and postpubertal growth phases, respectively, g_i_ was the total amount of gain (kg) in phase i ; b_i_ was the growth rate coefficient; t was age (wk); I_i_ was the inflection point (wk), or the age at which growth for phase i reached its maximum rate. The model was fitted to the Ross 708 breeder-recommended target BW to estimate the phase-specific BW gain coefficients in prepubertal, pubertal, and postpubertal growth phases. The BW gain coefficients in the prepubertal (g_1_) and pubertal (g_2_) growth phases were increased from 0% (CON) to 22.5% (CON+22.5%), in 2.5% increments to create 10 unique growth trajectories.

A completely randomized controlled study was conducted to relax the Ross 708 broiler breeder recommended growth trajectory. We fitted a 3-phase Gompertz growth model to the breeder recommended target BW (Aviagen, 2016) to estimate the phase-specific BW gain coefficients in prepubertal, pubertal, and postpubertal growth phases. The model had the form (Zuidhof, 2020):

$$BW_{t}=\sum_{i=1}^{i=3}g_{i}exp^{-exp^{-b_{i}\left(t-I_{i}\right)}}+\varepsilon_{t}$$

where BW_t_ was BW (kg) at time t (wk); g_i_ was the total amount of gain (kg) accruing in phase i; b_i_ was the growth rate coefficient for the i^th^; t was age (wk); I_i_ was the inflection point (wk), or the age at which growth for phase i reached its maximum rate; and ε_t_ was the residual error with an expected value of 0, and a normally distributed variance estimated by the software ε_t_ ∼ N(0,SD^2^); i was the growth phase (i = 1 to 3) where phase 1, 2, and 3 corresponded roughly to prepubertal, pubertal, and postpubertal growth phases, respectively. The BW gain coefficients in the prepubertal (g_1_) and pubertal (g_2_) growth phases were increased from 0% (**CON**; the breeder recommended target BW) to 22.5% higher (CON+22.5%), in 2.5% increments to create a total of 10 growth trajectories (Figure 1). A total of 36 broiler breeder pullets were used, in which 30 pullets were randomly assigned to one of the growth trajectories and 6 birds were assigned to an unrestricted group. The BW trajectories were applied to each individual bird using the PF system. Therefore, each bird was an experimental unit. The unrestricted group was not limited to a maximum BW and fed every time they used the PF stations.

The cloacae of all hens were palpated to detect hard-shelled eggs in the shell gland from 20 wk onward. Presence or absence of a hard-shelled egg in the shell gland was recorded daily for each bird to determine age at first egg (**AFE**). In the current study, birds came to lay at different ages due to being reared on various BW trajectories, creating a range for AFE criteria. In our previous publication, it was concluded that AFE advanced by 10.8 d per kg increase in photostimulation BW (Zukiwsky et al., 2021). Individual bird AFE ranged from 141 to 186 d of age with a median value of 175 d of age. In the current study 6 birds with lowest AFE were considered as the early onset of lay group (lower extreme of AFE), and 6 birds with highest AFE were considered as the late onset of lay group (upper extreme of AFE). The late onset of lay group included birds from the CON, CON+2.5%, CON+5%, CON+10%, and CON+12.5% treatments, and the early onset of lay group included birds from the CON+15%, CON+17.5%, CON+20%, and unrestricted treatments. The median BW of the multiple BW observations for individual birds at 154 d of age (22 wk) was considered as the photostimulation BW. The photostimulation BW of the candidate birds ranged from 2,350 g (CON group) to 4,940 g (unrestricted group) with a median value of 2,675 g. Thus, birds with a photostimulation BW greater than the median value were considered as the high BW group and those with a photostimulation BW lower than the median value were considered as the low BW group. We used AFE and photostimulation BW to create experimental classifications for the current CORT study. Both classification methods resulted in the same grouping. Thus, 2 experimental treatments (high vs. low BW) were compared in terms of the plasma CORT concentration.

### Plasma Sample Preparation

At 18, 20, 22, 24, and 26 wk of age, blood samples (3 mL) were collected from the brachial vein of all birds using a 4 mL sodium heparin vacutainer. The birds were restrained using a wired pen around a drinker, so the birds had access to water before blood collection. Then the birds were caught randomly from the pen and restrained gently to minimize the stress during blood collection. The blood samples were collected 1 to 3 h after onset of photophase in the morning and were immediately centrifuged at 1,244 × *g* at 4°C for 15 min to recover plasma. The plasma samples were stored at –20°C prior to CORT measurements.

### Plasma Corticosterone Determination Using ELISA method

Plasma samples were thawed on ice, and CORT concentration was determined using a commercially available CORT ELISA kit (Cayman Chemical, Ann Arbor, MI) according to the manufacturer's instructions. This kit is a competitive ELISA that can be used for quantification of CORT in plasma and fecal samples. The antibody in the kit specifically reacts with CORT and has less than 1% cross-reactivity with other adrenal hormones (Li et al., 2007). Briefly, the CORT ELISA standards were prepared in 9 dilutions ranging from 8.2 pg/mL to 50 ng/mL. Thawed plasma samples were vortexed and 50 µL of the samples were added in duplicate in individual wells of microtiter plates precoated with mouse anti-rabbit IgG antibody. Subsequently, 50 µL of CORT-acetylcholinesterase (**AChE**) conjugate (CORT Tracer) and 50 µL of CORT ELISA antiserum were added to the wells. Because the concentration of the CORT tracer was held constant while the concentration of CORT in the plasma samples varied, the amount of CORT tracer that was able to bind to the CORT antiserum would be inversely proportional to the concentration of CORT in the well (sample). The antiserum-CORT (either free or tracer) complex binds to the mouse anti-rabbit IgG that has been previously attached to the well. Thereafter the plate was washed to remove any unbound reagents and then Ellman's reagent (which contains the substrate to AChE) was added to the well. The product of this enzymatic reaction has a distinct yellow color and absorbs strongly at 412 nm. The intensity of this color, determined spectrophotometrically, was proportional to the amount of CORT tracer bound to the well, which was inversely proportional to the amount of free CORT in the well (originated from the plasma samples) during overnight incubation at 4°C. The inter- and intra-assay coefficients of variation were 6.2 and 10.9%, respectively.

### Plasma Corticosterone Determination Using LC-MS/MS Assay

#### Sample Preparation

Plasma samples were thawed on ice, in dark, before use. Then 100 µL of the samples (PBS as blank sample, calibration standards, quality control standards, and plasma samples) was mixed with 20 µL of internal standard mixture solution and were pipetted into Eppendorf tubes. After that, 100 µL of PBS buffer was added to each tube and vortexed for 30 s. Then 1,000 µL of methyl tert-butyl ether was added to each tube for extraction. The samples were shaken for 15 min. Subsequently, samples were centrifuged at 13,000 × *g*-force and 4°C for 15 min, and 750 µL of supernatants were transferred into HPLC vials and dried under nitrogen purge until completely dry. To the dried tubes, 100 µL of derivatization solution (1.5 M Hydroxylamine in HPLC grade water) was added, followed by shaking at 150 rpm for 15 min. All the tubes were then incubated at 60°C for 1 h, and subsequently 20 µL was injected into an UHPLC-equipped 4000 QTrap mass spectrometer (Sciex Canada, Concord, ON, Canada) for LC-MS/MS analysis.

#### LC-MS/MS Method

An Agilent 1260 series UHPLC system (Agilent, Palo Alto, CA) was used for LC-MS/MS analysis with an AB Sciex 4000 QTrap mass spectrometer. The controlling software for the LC-MS system was Analyst 1.5.2. For the HPLC work, solvent A was 0.1% formic acid in water; and solvent B was 0.1% formic acid in methanol. The gradient profile for the UHPLC solvent run was set as follows: t = 0 min, 10% B; t = 1.50 min, 10% B; t = 2.50 min, 55% B; t = 7.50 min, 95% B; t = 8.50 min, 95% B; t = 8.60 min, 10% B; and t = 12.0 min, 10% B. The flow rate was 0.5 mL/min and the sample injection volume was 20 μL. The mass spectrometer was set to a positive electrospray ionization mode with multiple reaction monitoring. The Ion Spray voltage was set at 5,500 V and the temperature at 550°C. The curtain gas, ion source gas 1, ion source gas 2, and collision gas were set at 40, 60, 60 and medium, respectively. The entrance potential was set at 10 V. Likewise, the decluttering potential, collision energy, collision cell exit potential, multiple reaction monitoring Q1 and Q3 were set individually for each analyte and internal standards.

### Statistical Analysis

The Pearson correlation coefficient was calculated using the CORR procedure of SAS (version 9.4; SAS Inst. Inc., Cary, NC), to measure the strength of the linear relationship between the plasma CORT measures using the ELISA and LC-MS/MS methods. The Pearson correlation coefficient “r” ranges from –1 to 1, where r = –1 indicates a perfect negative linear relationship, and r = 1 indicates a perfect positive linear relationship. The Pearson correlation was reported as significant where *P* ≤ 0.05.

Two-way analyses of variance were conducted using the MIXED procedure of SAS, with age and photostimulation BW as sources of variation. To account for correlated repeated measures, age was included in the model as a random effect, with individual birds as subjects. Pairwise differences between means were determined with the PDIFF option of the LSMEANS statement and were reported as significant when *P* ≤ 0.05.

## Results and Discussion

### Correlation Between ELISA and LC-MS/MS Methods

**Figure 2:**
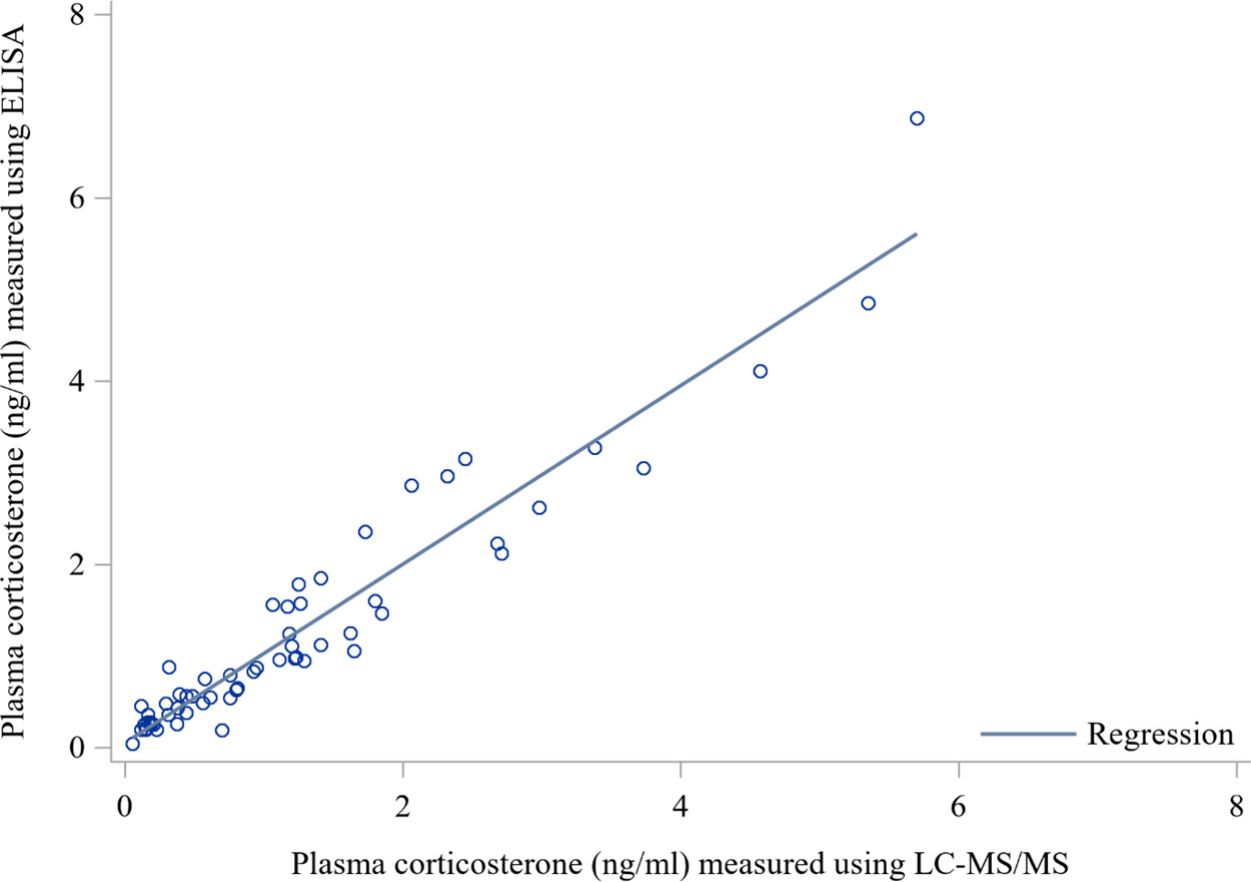
Linear relationship between plasma corticosterone (CORT) measures using ELISA and liquid chromatography-tandem mass spectrometry (LC-MS/MS) methods in heavy and light BW broiler breeders. Regression equation was: Plasma CORT (ng/mL) measured using ELISA = 0.05 + 0.97 Plasma corticosterone (ng/mL) measured using LC-MS/MS. R^2^ = 0.91 and Pearson correlation coefficient = 0.95.

Plasma CORT levels measured by the ELISA and LC-MS/MS methods were positively correlated (r = 0.95; *P* < 0.001). The slope (0.97) of the regression between ELISA and LC-MS/MS measures of plasma CORT indicated a high degree of agreement between the 2 assays (Figure 2). Thus, these methods can be used interchangeably to measure the plasma CORT in birds.

### Plasma Corticosterone Concentration

There was no effect of photostimulation BW or age on the plasma concentration of CORT (Table 1). In our previous publication we concluded that different feeding levels among the growth treatments created a range of photostimulation BW. More specifically, from 2 to 27 wk of age, ADFI increased in a range of 5.5 to 41.5 g per kg increase in photostimulation BW (Zukiwsky et al., 2021). Some previous research indicated elevation of plasma CORT levels attributed to increased feed restriction in broiler breeders (de Beer et al., 2008; Mormėde et al., 2007). Aranibar et al. (2020) compared the effect of skip a day feeding program and feeding broiler breeders soybean hulls on the off-feed day in a skip a day feeding program on concentration of plasma CORT. Their results showed that plasma concentration of CORT was greater in both groups 48 h after consuming the on-day feed amount compared to that of measured at 24 h after feeding. The authors concluded that the degree of feed restriction and the length of the fasting period between feedings had the most influence on plasma CORT level. Food restriction or starvation increased the mean glucocorticoids levels in humans and rats (Garcia-Belenguer et al., 1993; Kenny et al., 2014). Recently Manu et al. (2020) investigated saliva cortisol responses to feeding frequency in pregnant sows under isocaloric intake. The authors concluded that as all treatments groups had similar energy intake per kg live metabolic BW, splitting the limited feed from 1 meal into 2 or 3 meals and feeding multiple times within the day did not alter the basal cortisol concentrations in pigs. Although in the current study the birds were raised under different degrees of growth restriction programs, the individual's daily feeding frequency was greater than the conventional one-time meal per day. The results of daily meal frequency have been shown in our previous article (Zukiwsky et al., 2021). Briefly, the meal frequency for the restricted and unrestricted groups increased over a range of 1.8 to 5.8 meals per kg increase in photostimulation BW from 2 to 37 wk of age. Therefore, it can be concluded that the PF system provided frequent meals per day, so that the length of fasting between meals was not long enough to affect the plasma CORT level. More research is warranted to investigate the effect of feeding intervals on broiler breeder plasma CORT in a controlled experiment.

**Table 1:** Plasma concentration of corticosterone (ng/mL) measured by ELISA and liquid chromatography-tandem mass spectrometry (LC-MS/MS) methods in high and low BW broiler breeders.^1^

	ELISA					LC-MS/MS			
	High BW		Low BW			High BW		Low BW	
Age (wk)	Mean	SEM	Mean	SEM		Mean	SEM	Mean	SEM
18	1.46	0.61	2.40	0.97		1.50	0.66	2.20	0.76
20	0.73	0.38	0.49	0.18		0.72	0.45	0.50	0.20
22	2.03	0.70	1.21	0.44		2.08	0.78	1.18	0.54
24	1.39	0.38	0.58	0.21		1.47	0.35	0.49	0.14
26	1.26	0.44	1.06	0.23		1.23	0.46	1.06	0.16
Sources of variation	*——————————————— P*-value ————————————————								
Age	0.14					0.20			
BW	0.49					0.33			
Age×BW	0.64					0.49			

1 The median photostimulation BW of the candidate birds for measuring plasma corticosterone (CORT) concentration was used to define the upper (the high BW group) and lower (the low BW group) extremes.

To alleviate the intensity of feed restriction in broiler breeders, various degrees of relaxed growth targets were applied on pullets using the PF system. No effects of photostimulation BW or breeder age were observed on plasma CORT concentration as an index of animal welfare. Although some studies indicated that feed restriction can act as a stress factor leading to increase blood CORT (Mench, 1991; Hocking et al., 1996; de Beer et al., 2008; van Krimpen and de Jong, 2014), recent studies have attributed the changes in plasma CORT level to differences in metabolic rate as well as differences in level of stress ( Jimeno et al., 2020; Arrazola et al., 2019). A question that comes to mind is whether increased plasma CORT concentrations in feed restriction studies resulted from psychological and behavioral stress, metabolic stress, or both ( D’Eath et al., 2009). Some studies concluded that high levels of feed restriction require CORT regulation for glucose homeostasis during off-feed days in broiler breeders ( Arrazola et al., 2019; Aranibar et al., 2020). Further research is required to elucidate the underlying mechanisms of the relationship among feed restriction, plasma CORT level, and metabolic rate in breeders. A possible approach to do this would be using different levels of feed restriction and various levels of dietary energy and protein using a multifeeder PF system. An important consideration during blood collection is reducing blood sampling-associated stress, which could influence plasma CORT levels and interfere with or mask treatment effects. To minimize interfering with the animal at the time of blood sampling, we recognize that using automated blood sampling technique through a vascular catheter would be more ideal, but presents its own logistical and potentially welfare challenges with free run birds. This technique has been validated for use in rats, with negligible impact on stress-associated hormone levels ( Abelson et al., 2005; Siswanto et al., 2008), but it needs to be validated in poultry as well. Furthermore, diurnal variations in plasma CORT levels have been reported in several studies on laying hens (Johnson and van Tienhoven, 1981), broiler chickens ( Lauber et al., 1987), broiler breeder pullets (Aranibar et al., 2020; Arrazola et al., 2019), and turkeys (Proudman, 1991). These variations were associated with feeding time, which is not particularly relevant to the current study where birds were fed sequentially at any time of day or night, using a precision feeding system. In the current study, blood samples were taken from each bird between 1 and 3 h after the start of the photoperiod, which may have interfered with nesting behavior. However, any temporal effects on plasma CORT levels are assumed to have been similar across all treatments. The results of the current study indicated highly positive correlation between CORT measures using ELISA and LC-MS/MS methods. This suggests that both methods are valid for measuring plasma CORT.
